# The Avalanche Hypothesis and Compression of Morbidity: Testing Assumptions through Cohort-Sequential Analysis

**DOI:** 10.1371/journal.pone.0123910

**Published:** 2015-05-11

**Authors:** Jordan Silberman, Chun Wang, Shawn T. Mason, Steven M. Schwartz, Matthew Hall, Jason L. Morrissette, Xin M. Tu, Janet Greenhut

**Affiliations:** 1 School of Medicine and Dentistry, University of Rochester Medical Center, Rochester, New York, United States of America; 2 Wellness & Prevention, Inc., Ann Arbor, Michigan, United States of America; 3 School of Medicine, Johns Hopkins University, Baltimore, Maryland, United States of America; 4 SocialWellth, Inc., Las Vegas, Nevada, United States of America; 5 Highmark, Inc., Pittsburgh, Pennsylvania, United States of America; 6 Department of Biostatistics and Computational Biology, University of Rochester Medical Center, Rochester, New York, United States of America; 7 Center of Excellence at Canandaigua, Canandaigua VA Medical Center, Canandaigua, New York, United States of America; Örebro University, SWEDEN

## Abstract

**Background:**

The compression of morbidity model posits a breakpoint in the adult lifespan that separates an initial period of relative health from a subsequent period of ever increasing morbidity. Researchers often assume that such a breakpoint exists; however, this assumption is hitherto untested.

**Purpose:**

To test the assumption that a breakpoint exists—which we term a morbidity tipping point—separating a period of relative health from a subsequent deterioration in health status. An analogous tipping point for healthcare costs was also investigated.

**Methods:**

Four years of adults’ (*N* = 55,550) morbidity and costs data were retrospectively analyzed. Data were collected in Pittsburgh, PA between 2006 and 2009; analyses were performed in Rochester, NY and Ann Arbor, MI in 2012 and 2013. Cohort-sequential and hockey stick regression models were used to characterize long-term trajectories and tipping points, respectively, for both morbidity and costs.

**Results:**

Morbidity increased exponentially with age (*P*<.001). A morbidity tipping point was observed at age 45.5 (95% CI, 41.3-49.7). An exponential trajectory was also observed for costs (*P*<.001), with a costs tipping point occurring at age 39.5 (95% CI, 32.4-46.6). Following their respective tipping points, both morbidity and costs increased substantially (*Ps*<.001).

**Conclusions:**

Findings support the existence of a morbidity tipping point, confirming an important but untested assumption. This tipping point, however, may occur earlier in the lifespan than is widely assumed. An “avalanche of morbidity” occurred after the morbidity tipping point—an ever increasing rate of morbidity progression. For costs, an analogous tipping point and “avalanche” were observed. The time point at which costs began to increase substantially occurred approximately 6 years before health status began to deteriorate.

## Introduction

**Fig 1:**
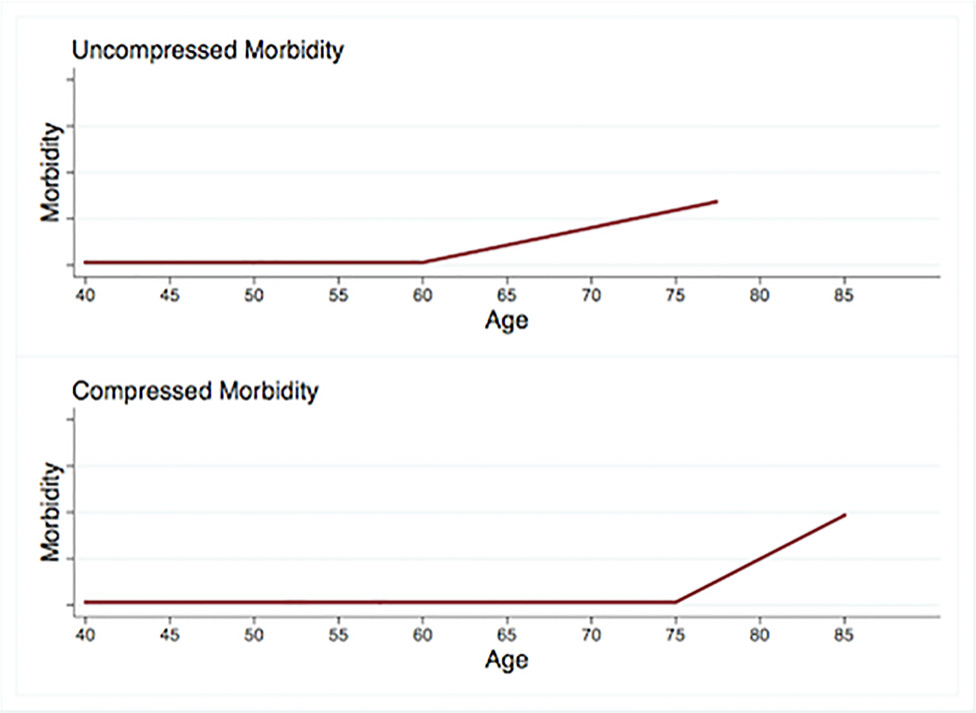
Compression of morbidity. Relative to the top trajectory, the bottom trajectory shows a 15-year delay in morbidity onset and a 7.5-year delay in death. Because the morbidity onset delay exceeds the delay in age of death, the morbidity period shown in the lower trajectory is compressed.

For more than 30 years, the compression of morbidity (COM) model has provided a framework for research programs in epidemiology, physiology, gerontology, and other fields [1–4]. First proposed by Fries and colleagues in 1980 [5], the COM model suggests that a) both onset of morbidity and age of death can be delayed through changes in behavior, b) behavior change has the potential to delay morbidity onset for longer than it can delay mortality, and c) healthy behavior therefore has the potential to decrease the length of time at the end of the lifespan that is marked by substantial morbidity (see Fig 1) [2].

It is assumed, under the COM model, that a breakpoint exists in the morbidity trajectory—a time point in the adult lifespan distinguishing an initial period of relative health from a subsequent period marked by ever increasing physiological dysfunction [2]. The existence of such a time point is essential to the COM paradigm. Indeed, without a time point delineating premorbidity from a period of morbidity escalation, it would not be possible to quantify durations of premorbid and morbid time periods, nor would it be possible to identify compression in the length of time marked by escalating morbidity. It is not surprising, then, that Fries [6] posits a time point separating a premorbid time period from a subsequent “crescendo of chronic disease.”

Given that a morbidity breakpoint is an important tenet of the COM model, and given the large literature on compression of morbidity [2,4], it is somewhat surprising that investigators have not empirically demonstrated the existence of a breakpoint in the morbidity trajectory. One feasible approach to investigating the hypothesized breakpoint is through cohort-sequential (CS) analysis (described below). In the present work, we use cohort-sequential methods to test the assumption that a definable breakpoint exists, separating a period of relatively good health from a subsequent deterioration in health status. It is often assumed in the compression of morbidity literature that such a breakpoint exists; however, to the best of our knowledge, this assumption is hitherto untested.

### The Avalanche Hypothesis and the Morbidity Tipping Point

Following others [1], we define morbidity as the “physiological dysregulation, disease onset, functioning loss, and frailty” that typically occur at some time during adulthood. The avalanche hypothesis (AH) posits that, after a definable time point, there is a profound change in the manner in which morbidity unfolds over time. The AH does not merely suggest that morbidity progresses faster after this time point. Rather, the AH posits that, after this time point, there is a sharp increase in the rate of change *of the rate of change* in morbidity. The AH predicts that a) before this time point, morbidity progresses at a slow, constant rate and b) an ever increasing rate of morbidity progression is observed after the aforementioned time point. Following this time point, morbidity begins spiraling out of control, such that the increase in morbidity observed during each year tends to exceed that of the preceding year. We refer to this point in the adult lifespan as the morbidity tipping point (MTP).

The avalanche hypothesis can be conceptualized both mathematically and metaphorically. Expressed mathematically, the AH states that the second derivative of morbidity with respect to age increases significantly at the morbidity tipping point. Expressed metaphorically, the AH suggests that morbidity progression observed during adulthood is analogous to the progression of an avalanche.

The “pathogenesis” of an avalanche begins when snow accumulates in a precarious location. Gradually, the snow mound grows into a large, unstable structure. After reaching a threshold mass, the mound destabilizes and sweeps down a mountainside. Shortly after it starts, the avalanche accelerates, such that the speed observed at a given moment exceeds that of the preceding moment.

We hypothesize that morbidity progression is analogous to the development and triggering of an avalanche. In early stages, an unstable snow mound has no perceivable effects and grows imperceptibly slowly; therefore, it often goes unnoticed until an avalanche occurs. Early stage morbidity, analogously, often progresses slowly, and goes unnoticed until symptoms suddenly intensify. We hypothesize that, like an avalanche, morbidity reaches a tipping point at which it begins to progress at ever increasing rates.

The aforementioned morbidity trajectory was hypothesized on the basis of both empirical evidence and anecdotal observation. Evidence suggesting this trajectory comes from research indicating that, as people age, the degree of physiological dysfunction caused by a given challenge to homeostasis increases exponentially [6,7]. This exponential pattern suggests that, relative to young adults, older adults may show faster rates of change *of rates of change* in morbidity. Anecdotally, moreover, we find that patients often reach a point at which morbidity spirals out of control—a point after which every passing year brings with it a new pathology that is far more severe than those of the preceding year.

### An Analogous Trajectory for Healthcare Costs

In addition to hypothesizing a tipping point and subsequent “avalanche” for morbidity, we also hypothesized the same trajectory for healthcare costs. Because individuals often seek healthcare after experiencing symptoms, we expected the tipping point for costs to occur shortly after that of morbidity.

### Objectives of the Present Investigation

The key objectives of this work were twofold. First, we sought to test the hypothesis that a morbidity tipping point exists in the adult lifespan, delineating an initial period of relative health from a subsequent deterioration in health status. It is important to test this hypothesis because the existence of such a tipping point is assumed throughout the sizable compression of morbidity literature, but this assumption is hitherto untested. Moreover, if a morbidity tipping point exists, then it could be useful for compression of morbidity research. The morbidity tipping point may provide a meaningful, quantifiable distinction between premorbidity and morbidity, allowing COM researchers to better quantify compression in durations of time periods marked by morbidity escalation.

The second objective of this work was to provide a model that would facilitate testing of the hypothesized morbidity tipping point. Thus, the second objective was subservient to the first. Without a model that makes testable predictions regarding differences between morbidity observed pre-MTP and those observed post-MTP, it would not be possible to statistically test the hypothesis that a morbidity tipping point exists. The avalanche hypothesis offers one such a model, positing specific, quantifiable differences between pre-MTP and post-MTP time periods. The AH provides a conceptual and quantitative framework through which the existence of a morbidity tipping point can be tested.

To these ends, we analyzed trajectories for a proxy measure of morbidity (described below) that were observed throughout adulthood in a large population. Costs trajectories were analyzed as well.

We made 4 predictions regarding trajectories of morbidity and costs. First, we predicted that morbidity would increase exponentially with age. Second, we predicted that a morbidity tipping point would be observed at some time during adulthood. Third, we predicted an exponential trajectory and tipping point for costs mirroring the trajectory and tipping point of morbidity. Our final prediction was that the costs tipping point would be observed after the tipping point for morbidity.

## Methods

### Design

A retrospective design was used to investigate trajectories of morbidity and healthcare costs. Data were provided by a large Western Pennsylvania health insurer. Cohort-sequential analysis [8–13] and hockey stick regression [14,15] (both are described below) were used to test the aforementioned predictions. An independent institutional review board (Allendale Investigational Review Board) approved this study. Consent was not obtained because data were analyzed anonymously and reported only at highly aggregated levels of analysis.

### Study Sample

The sample was comprised of 55,550 members of Highmark, Inc., a Blue Cross Blue Shield health plan. Mean (SD) age at baseline was 44.7 (9.2), and the sample was 51.1% female. Race and ethnicity data were not available.

### Exclusion Criteria

**Fig 2:**
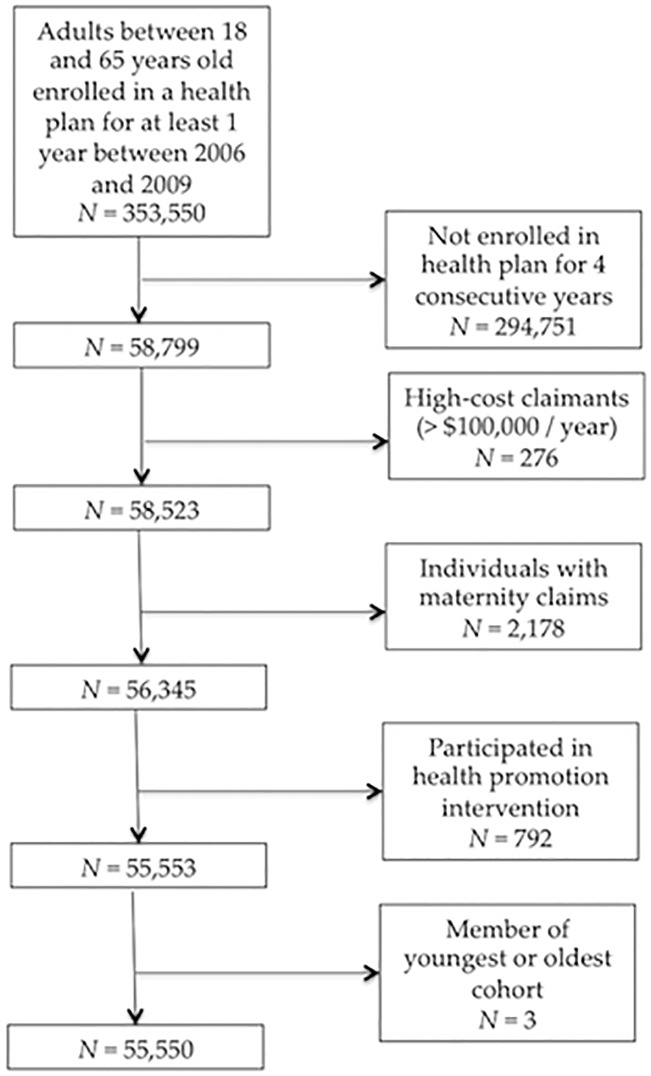
Effects of exclusion criteria on sample size.

Prospective subjects were filtered per the following exclusion criteria (see Fig 2). We excluded those who were not enrolled with the insurer for the 4 consecutive years between 2006 and 2009, the 4 years for which data were available. High-cost claimants (> $100,000 in a single year) were excluded to avoid the disproportionate impact of cost outliers who required unusually expensive healthcare services. Those with maternity claims were excluded because healthcare services related to maternity are not relevant to the predictions investigated herein. Study hypotheses pertain to natural histories of morbidity and costs, in the absence of intervention; subjects who had participated in a health promotion program were therefore excluded. Finally, the youngest (age 18 at baseline) and oldest (age 62 at baseline) age cohorts had to be excluded because the small sizes of these cohorts (just 2 subjects and 1 subject, respectively) caused within-cohort variances that were too low to estimate cohort-sequential models.

### Outcome Variables

Diagnostic cost group scores (DxCGs) [16] were used as a proxy measure of morbidity. This measure reflects overall health status. One DxCG score was computed for each subject, for each year between 2006 and 2009, using DxCG RiskSmart® software 2.11 (Verisk Health Inc., Waltham, MA).

Costs data were obtained from insurance records; these data reflect allowed payments per person per calendar year. Dollar amounts were adjusted to 2009 values using Consumer Price Indices of the U.S. Bureau of Labor Statistics [17].

Of note, the STROBE (Strengthening the Reporting of Observational Studies in Epidemiology) Statement [18] for reporting observational studies suggests that alternative methods that could have been used to study age-related trends in morbidity and costs should be discussed. We therefore note that alternative approaches to collecting data for the present investigation could have included surveying adults with validated self-report health questionnaires, collecting diagnostic data from electronic medical records, extracting billing information from other payers’ databases, etc.

### Aggregation of Multiple Age Cohorts with Cohort Sequential Analysis

Cohort-sequential models were used to estimate trajectories for morbidity and annual healthcare costs between 19 and 64 years of age. The CS design can be conceptualized as several simultaneous longitudinal studies, each relatively short in duration, that are statistically aggregated to estimate long-term growth curves. Evidence suggests that results of CS methods are consistent with those of traditional longitudinal research [10–12,19,20]. Further details regarding CS models are presented elsewhere [8–13]. CS designs are useful when hypotheses pertain to long time periods, but available resources (financial or otherwise) can support data collection only for relatively short time periods. Hypotheses described herein pertain to the entire adult lifespan. One approach to investigating these hypotheses would have involved traditional longitudinal research; however, this would have taken roughly half a century and cost tens of millions of dollars. Such an undertaking was infeasible, so cohort-sequential analysis was used.

For the present study, CS models served two purposes. First, they allowed us to determine whether or not it was appropriate to aggregate data from multiple age cohorts into a single, common trajectory. Second, CS models allowed us to test the hypotheses that morbidity and costs increase exponentially with age.

**Fig 3:**
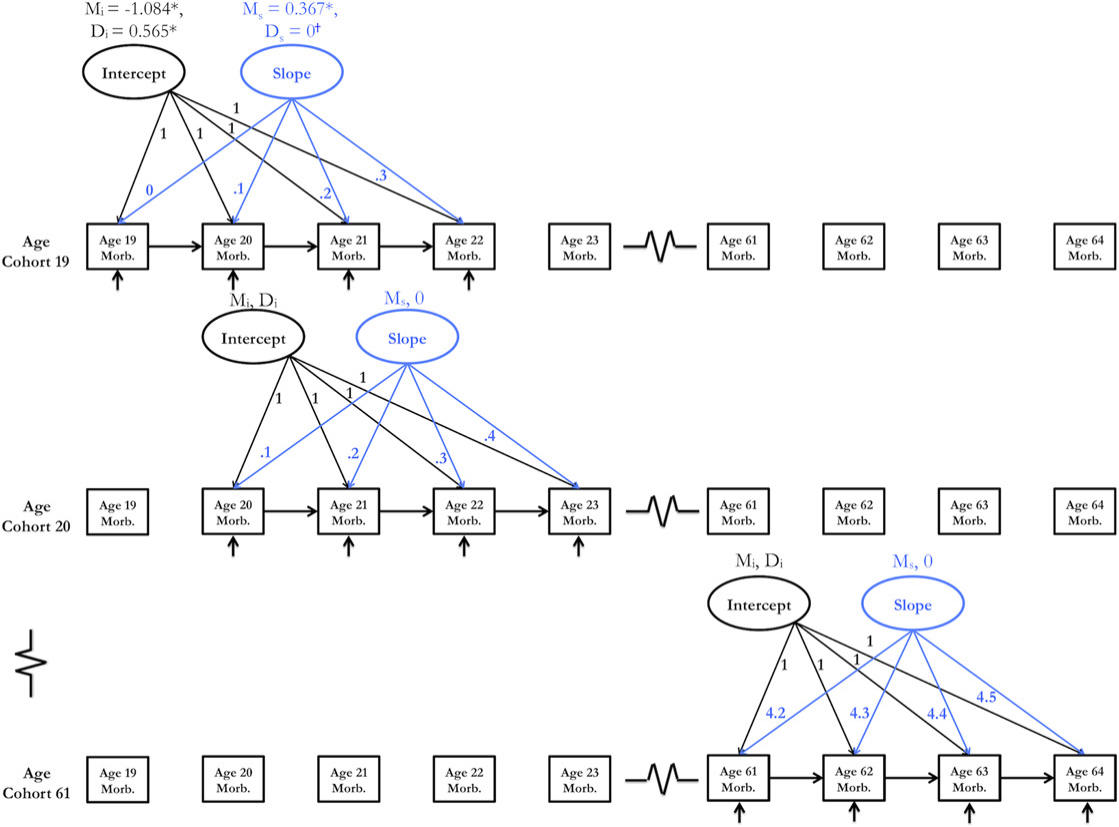
Cohort-sequential model for morbidity. Model fit was adequate (χ^2^(508) = 749.078, *P*<.001; χ ^2^/*df* [NC] = 1.475; CFI = .997; RMSEA = .019). M_i_ was constrained to equality across age cohorts, as were D_i_ and M_s_. Autoregressive pathway coefficients are listed in S1 Table. Asterisks indicate *P*<.001; dagger, parameter constrained to 0; M_i_, mean intercept; D_i_, intercept variance (disturbance term); M_s_, mean slope; D_s_, slope variance; Morb., morbidity.

Two CS models were estimated—one for morbidity and one for costs (Fig 3 and S1 Fig). Mplus 6.12 (Muthén & Muthén, Los Angeles, CA) was used to estimate both cohort-sequential models. Each model estimated a single trajectory, across all age cohorts, describing change over time in outcome (morbidity or costs) between 19 and 64 years of age. Models were specified such that they could be used to test for the presence of exponential trajectories in outcomes.

### Specification of Cohort-Sequential Models

Natural log-transformed morbidity scores were modeled as a function of age, as well as an autoregressive coefficient. Thus, the CS model estimating morbidity at a given age could be written as follows: ln(morbidity) = intercept + b_a_(age) + b_ar_(ln(morbidity)_(age-1)_), where b_a_ and b_ar_ are age and autoregressive coefficients, respectively, and ln(morbidity)_(age-1)_ represents the natural log of the morbidity score observed one year prior. This can of course be rewritten as the following exponential model: morbidity = *e* ^*(intercept* + *b*^_a_^(age) + *b*^_ar_^(ln(morbidity)^_(age-1)_^))^. Thus, adequate model fit in combination with a significant age coefficient would suggest that morbidity increases exponentially as age increases linearly. This allowed us to test the hypothesis that morbidity increases exponentially as a function of age.

An analogous approach was used to investigate the hypothesized exponential trajectory for costs. All costs values were adjusted to 2009 dollars using the Consumer Price Indices from the U.S. Bureau of Labor Statistics [17], and were natural log-transformed. The resultant values were specified as the outcome variable in the cohort sequential model for costs. Predictors specified in this model were identical to those of the aforementioned morbidity model.

We specified 3 years of overlap between adjacent age cohorts—the maximum amount of overlap possible (see Fig 3)—because maximizing overlap between adjacent cohorts may minimize the likelihood of discrepancies between CS models and true longitudinal models [21]. CS models were censored from below to account for floor effects (large numbers of subjects had very low morbidity scores and/or annual healthcare costs) [22]. Inclusion of autoregressive coefficients reflects the assumption that outcome scores observed in a given year should predict outcome scores observed in the following year. Random slopes were excluded (ie, slope variances were constrained to 0) because their inclusion prevented model convergence. Thus, it was not possible to estimate intercept-slope covariances.

Slope loadings pertaining to a given age were constrained to equality across overlapping age cohorts. For example, as shown in Fig 3, the slope loading for age 20 is constrained to equal 0.1 for both age cohort 19 and for age cohort 20. Analogous constraints were specified for each age between 21 years and 63 years (such constraints were unnecessary for ages 19 and 64 because only the youngest and oldest included cohorts, respectively, were observed at these ages). Similarly, autoregressive pathways were constrained to equality across overlapping age cohorts for each pair of consecutive ages. Underlying these constraints is the assumption that overlapping age cohorts show the same trajectories—that the mean score for an outcome (morbidity or costs) should be the same for a given age, irrespective of the age cohort in which the mean is observed. Statistical verification of this assumption is necessary before one can appropriately aggregate data from multiple age cohorts into a single, unified trajectory [19]. A detailed rationale for other model specifications has been presented elsewhere [8,11,12].

### Identifying Morbidity Tipping Points through Hockey Stick Regression

After using CS models to confirm that it was statistically appropriate for data from multiple age cohorts to be aggregated into a single trajectory, hockey stick regressions (HSRs) [14,15] were used to test for the presence of tipping points. HSR identifies the optimal positions of breakpoints (also called “knots”) in piecewise regression models. Stata 12.1 (StataCorp, College Station, TX) was used to estimate HSRs.

Two HSRs were estimated—one for morbidity and one for costs. Following others [23,24], we used a two-step procedure in which a) outcome (morbidity or costs) was regressed on age separately for each subject, and b) the resultant unstandardized coefficients were hockey stick regressed on study subjects’ mean ages during the 4-year study period.

Regressing regression coefficients usually causes heteroskedasticity [25,26], and indeed, HSR models for both morbidity and costs were heteroskedastic (*P*s < .001, White’s general test) [27]. The feasible generalized least squares (FGLS) approach is recommended for estimating heteroskedasticity-robust regression models in which the dependent variable is a set of coefficients from previous regressions [25,26,28]. Note that weighted least squares (WLS) estimators are not recommended for achieving robustness to heteroskedasticity in such models, because the statistical performance of these estimators may be poor when regressing regression coefficients [28].

For the present hockey stick regression analyses, we chose an estimation method that is extremely similar to FGLS, namely, feasible generalized nonlinear least squares (FGNLS). The FGNLS estimator accounts for heteroskedasticity in the same manner as FGLS [29]. FGLS and FGNLS methods have been detailed elsewhere [29–31]. FGNLS was utilized because a) it accounts for the heteroskedasticity observed when regressing regression coefficients in the same manner as FGLS [29] and b) FGNLS estimation could be feasibly implemented for hockey stick regression models. Stata’s **nlsur** command in conjunction with the **fgnls** option were used to estimate hockey stick regressions with the feasible generalized nonlinear least squares estimator. The approach used was identical to one recommended by Mitchell [32] for estimating hockey stick regressions, except that the heteroskedasticity-robust FGNLS method was used rather than ordinary least squares estimation (Stata syntax is available from the first author upon request).

Coefficients specified as dependent variables in HSRs reflected rates of change. A breakpoint identified in one of these HSRs indicated a slope fluctuation in a regression of change scores on age. Thus, a breakpoint would indicate a tipping point—a significant increase or decrease in the rate of change of the rate of change.

### Missing Data

Missing data in the cohort-sequential model for morbidity were handled as follows. Subjects who had missing morbidity scores for all 4 study years (less than 0.1% of the sample) were excluded. Missing morbidity scores were expectation-maximization imputed [33] for subjects missing scores pertaining to 1 of the 4 study years (less than 0.5% of the sample) or 2 of the 4 study years (less than 0.1% of the sample). No subject had 3 missing morbidity scores and no age data were missing.

The hockey stick regression model for morbidity excluded any subject for whom morbidity scores were not available at all 4 time points (less than 1% of the sample). Thus, all regression coefficients representing subject-specific rates of change in morbidity were based on 4 observed morbidity scores.

Neither the cohort-sequential model for costs nor the hockey stick regression model for costs required us to address missing data. Costs data were available for all subjects, at all time points.

## Results

### Morbidity

**Fig 4:**
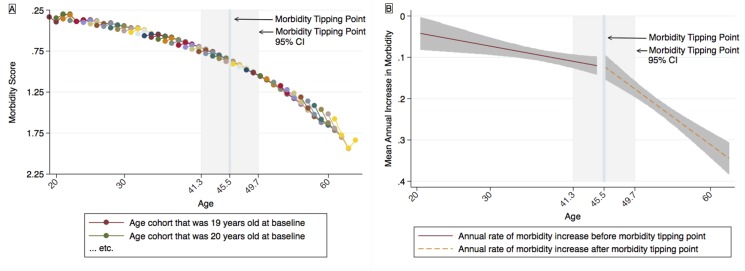
Model estimates for morbidity and rates of morbidity progression. A: Cohort-sequential model estimates for morbidity. B: Hockey stick regression model estimates for rates of morbidity progression. The tipping point indicates the estimated age at which a significant increase is observed in the rate of change *of the rate of change*. Model estimates are shown in their original metrics. Note that the y axis in Fig 4A represents model-estimated morbidity scores, while that of Fig 4B represents changes over time in morbidity. Thus, slopes shown in Fig 4A and 4B represent 1^st^ and 2^nd^ derivatives, respectively. Both y axes are reverse scaled, such that values positioned higher on the page indicate more favorable outcomes (better health status or slower morbidity progression). Shaded regions indicate 95% confidence intervals.

The cohort-sequential model for morbidity (Fig 3) showed adequate fit (χ^2^(508) = 749.078, *P*<.001; χ^2^/*df* [NC] = 1.475; CFI = .997; RMSEA = .019). Note that a χ^2^/*df* ratio less than 2, a CFI greater than .95, and an RMSEA less than .06 all indicate that the model fits observed data adequately [34]. Fit indices suggest that the model can be treated as if all age cohorts were sampled from the same population [9], and that data from multiple age cohorts can be aggregated into a common morbidity trajectory [19]. The mean slope and mean intercept were both significant (M_S_ = 0.367; 95% CI, 0.357–0.376; *P*<.001; M_i_ = -1.084; 95% CI, -1.111 to -1.056; *P*<.001). The significant mean slope supported the hypothesized exponential morbidity trajectory. The intercept variance was also significant (D_i_ = 0.565; 95% CI, 0.549–0.582; *P*<.001), a finding analogous to a significant random intercept in a mixed-effects model. Model estimated morbidity scores are shown in Fig 4A.

Having established that data could be aggregated into a single, exponential trajectory, we proceeded to estimate a hockey stick regression model. A morbidity tipping point was observed at 45.5 years of age (95% CI, 41.3–49.7; see Fig 4B). Slopes observed both before and after the MTP were significantly greater than 0 (*b* = 0.00319; 95% CI, 0.000394–0.00599; *P* = .03 and *b* = 0.0130; 95% CI, 0.00969–0.0163; *P*<.001, respectively). These slopes, moreover, were significantly different from each other (∆*b* = 0.00981; 95% CI, 0.00557–0.0141; *P*<.001; the significance of ∆*b* was tested using a method described elsewhere) [32].

### Healthcare Costs

**Fig 5:**
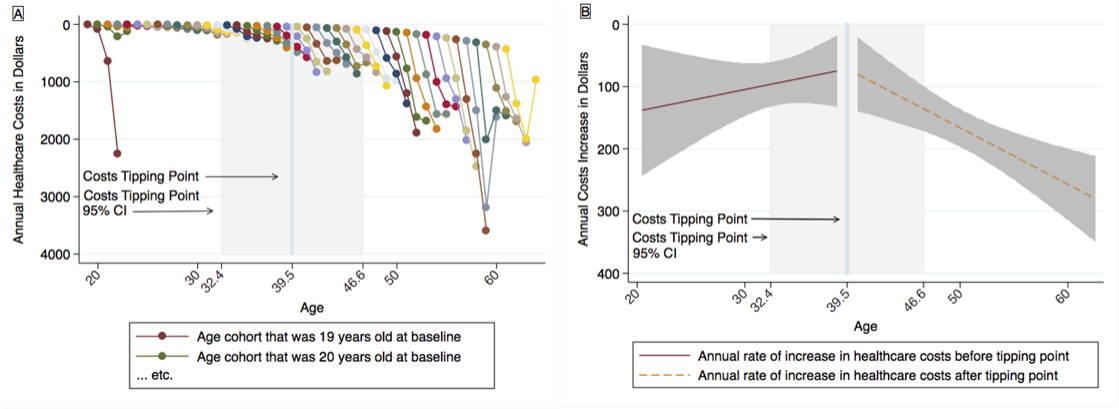
Model estimates for costs and for the rates at which costs increase. A: Cohort-sequential model estimates for costs. B: Hockey stick regression model estimates for rates at which costs increase. The tipping point indicates the estimated age at which a significant increase is observed in the rate of change *of the rate of change*. Model estimates are shown in their original metrics. Note that the y axis in Fig 5A represents annual cost estimates, while that of Fig 5B represents changes over time in costs. Thus, slopes shown in Fig 5A and 5B represent 1st and 2nd derivatives, respectively. Both y axes are reverse scaled, such that values positioned higher on the page indicate more favorable outcomes (lower costs or slower rates of growth in costs). Shaded regions indicate 95% confidence intervals. Cost estimates were adjusted to 2009 values using the Consumer Price Indices of the U.S. Bureau of Labor Statistics [17].

The cohort-sequential model for costs (see Fig 5A and S1 Fig) also showed adequate fit (χ^2^(508) = 568.953, *P* = .03; χ^2^/*df* [NC] = 1.120; CFI = 1.000; RMSEA = .010). Thus, we can again treat the data as if all observations were sampled from the same population [9], and aggregate costs data from multiple age cohorts into a single trajectory [19]. The mean slope and mean intercept were significant (M_S_ = 1.038; 95% CI, 0.775–1.301; *P*<.001; M_i_ = 1.731; 95% CI, 0.552–2.910; *P* = .004), as was the intercept variance (D_i_ = 2.043; 95% CI, 0.961–3.124; *P*<.001). Autoregressive pathway estimates for both CS models are presented in S1 Table.

We proceeded to estimate an HSR model for costs mirroring that of morbidity. A tipping point was observed at 39.5 years of age (95% CI, 32.4–46.6; see Fig 5B). Prior to this tipping point, the rate of change of annual increases in costs was not significant (*b* = -4.241; 95% CI, -14.424 to 5.942; *P* = .41). After this time point, in contrast, the rate of change of the rate of change was significantly greater than 0 (*b* = 9.569; 95% CI, 4.893–14.245; *P*<.001). The post-tipping point slope, moreover, was significantly greater than the slope observed before the costs tipping point (∆*b* = 13.810; 95% CI, 2.666–24.954; *P* = .02).

## Discussion

In the present study we analyzed trajectories for morbidity and costs observed throughout adulthood in a large sample. The key results were that a) morbidity increased exponentially with age, b) a morbidity tipping point was identified, delineating an initial period of relative health from a subsequent deterioration in health status, and c) an analogous exponential trajectory and tipping point were observed for healthcare costs. These three results were consistent with predictions. Contrary to predictions, the morbidity tipping point occurred 6 years *after* the tipping point for healthcare costs; the tipping points for morbidity and costs were observed at 45.5 years of age and 39.5 years of age, respectively.

As stated above, the key objectives of the present work were a) to test the widely-assumed but untested hypothesis that a morbidity tipping point occurs during the adult lifespan, and b) to provide a model that can serve as a conceptual and quantitative framework, through which the existence of a morbidity tipping point can be tested. Both study objectives were achieved. A significant increase was observed, at 45.5 years of age, in the rate of change *of the rate of change* in morbidity. The rate of increase in the speed of morbidity progression was relatively low before this tipping point, and was significantly greater after the MTP. Thus, findings support a crucial but hitherto untested assumption of the compression of morbidity literature—the assumption that a breakpoint separates an initial period of relative health from a subsequent period of escalating morbidity. In addition, findings suggests that this tipping point can be quantified in the context of the avalanche model, which posits a significant difference between the second derivative of morbidity observed before the MTP and that observed after the MTP.

### Why Might the Tipping Point for Costs Occur Before that of Morbidity?

Perhaps the most interesting finding generated from the present work was that, contrary to predictions, the tipping point for costs did not occur after that of morbidity. Rather, the costs tipping point occurred approximately 6 years *before* the MTP. One possible explanation for this is that preventive health services—which may increase short-term costs even in the absence of increased morbidity—caused costs to begin spiraling out of control before health status began to do so.

The hypothesis that preventive services costs explain the counterintuitive sequence of tipping points could be tested by controlling for preventive services costs in the model that was used to estimate the costs tipping point. If this hypothesis is correct, then a model that controls for preventive services costs should yield an attenuated tipping point (ie, a decrease in the degree to which the second derivative changes at the tipping point). Unfortunately, available data did not link costs to specific healthcare services; however, data were available for annual outpatient costs. Presumably, most preventive services fall into the outpatient category. It was therefore possible to use outpatient costs data to further explore this issue.

If we were to control for outpatient costs in the aforementioned costs HSR model, and if this attenuated the costs tipping point, then we would not know whether the change were due to a) costs of preventive health services, b) costs of non-preventive services that also fall into the outpatient category, or c) a combination of both. If, on the other hand, controlling for outpatient costs were to have no effect on the costs tipping point, and if we are willing to accept the assumption that most preventive services are administered in the outpatient setting, then we could conclude that neither preventive services costs nor any other type of outpatient costs accounts for the counterintuitive order of tipping points. Thus, controlling for outpatient costs might allow us to reject the hypothesis that preventive services costs account for the temporal position of the costs tipping point, but this analysis would not allow us to conclude confidently that the position of the costs tipping point was driven by preventive services. To potentially rule out preventive health services as a driver of the costs tipping point, a post-hoc analysis was performed (see S1 Text for details).

This post-hoc analysis revealed no attenuation of the costs tipping point after controlling for outpatient costs (see S1 Text). Thus, results of the post-hoc analysis were inconsistent with the hypothesis that preventive health services caused costs to begin spiraling out of control prior to the time at which health began to deteriorate.

Another candidate explanation for the temporal sequence of tipping points is that small area variations in standards of practice, poorly designed incentives, and other factors caused clinicians to routinely recommend unnecessary health services. Indeed, evidence suggests that providers may sometimes recommend services that yield little benefit [35–37]. This may decouple health status from level of healthcare provided. And, if there is no relationship between the need for and the provision of healthcare services, then we have no reason to expect any specific relationship between the temporal sequences of changes in these two variables. It is, of course, unlikely that health status and provision of healthcare services have become entirely independent. However, it may be the case that these two variables have become decoupled to a degree at which the temporal sequence arising from normal coupling of these variables has begun to become obscured. Decoupling the connection between the need for and the provision of healthcare services may in part explain the discrepancy between the age at which costs began to spiral out of control and the age at which health began to deteriorate. Further research is needed to investigate this possibility.

### Limitations

Range restriction of subject ages, lack of race and ethnicity data, and inclusion of only insured individuals, are all limitations of this study. Use of cohort-sequential methods rather than longitudinal methods may also be considered a limitation; however, as mentioned above, evidence suggests that results of cohort-sequential studies do not differ from those of true longitudinal studies [10–12,19,20]. Exclusion of those who did not have health insurance through the same insurer for 4 consecutive years (2006–2009) may also limit generalizability of results. Unfortunately, data pertaining to excluded individuals is not available for analysis. In addition, it is noteworthy that there is some overlap between the confidence intervals of the two tipping points; findings should therefore be interpreted with caution.

It may be useful to test whether or not early costs tipping points predict early MTPs. However, because each subject’s data span just 4 years, it was not possible to investigate this possibility. Future research is needed to explore this issue.

### Significance of the Present Work

Despite limitations, the present work may be important for at least 3 reasons. First, the age at which the morbidity tipping point occurred—45.5 years old—was substantially younger than what might be expected based on the COM literature. Many COM studies use somewhat arbitrarily defined morbidity cutoffs, and assume that a breakpoint exists during or after the fifth decade of life (examples can be found elsewhere) [2,6,38–41]. Thus, results suggest that much of the compression of morbidity literature may present misleading findings regarding the age at which health status begins to deteriorate.

Second, methods described herein provide one useful approach for identifying breakpoints between premorbidity and morbidity. This or similar approaches for quantitatively identifying meaningful breakpoints in the morbidity trajectory may be preferable to the somewhat arbitrary selection of breakpoints that is often observed in COM research. The unexpectedly young age at which the morbidity tipping point was observed in the present study underscores the importance of quantitatively identifying the age that best delineates premorbidity from morbidity. Identifying morbidity tipping points through quantitative methods may prevent COM researchers from overestimating the age at which deterioration in health status begins to occur. The methods described herein, moreover, may be useful for testing the existence of hypothesized tipping points in other variables across the lifespan. These methods may also be useful for testing other, analogous hypotheses that are difficult to investigate using standard methodologies. This may be a fruitful future direction.

Note that the method for identifying tipping points presented herein is just one of many possible approaches. Other quantitative approaches are certainly possible; future research is needed to investigate such approaches.

Finally, in addition to utility in COM research, the present findings may also be useful for guiding the provision of preventive services. Results suggest that the tipping point for costs may predict a looming tipping point for morbidity. It might be possible to exploit this pattern of trajectories. If costs data can be used to identify individuals for whom a morbidity tipping point is expected to occur within several years, then those individuals could be aggressively targeted with preventive health services. Using this approach, it may be possible to prevent or delay anticipated morbidity tipping points. Future research is needed to develop methods for predicting morbidity tipping points from costs data, and, if possible, to determine how targeted preventive services can be utilized most effectively to delay or prevent MTPs and the subsequent “avalanche” of morbidity.

## Supporting Information

S1 Fig Cohort-sequential model for annual healthcare costs. Model fit was adequate (χ2(508) = 568.953, *P* = .03; χ2/*df* [NC] = 1.120; CFI = 1.000; RMSEA = .010). Note that a χ2/df ratio less than 2, a CFI greater than .95, and an RMSEA less than .06 all indicate that the model fits observed data adequately.1 M_i_ was constrained to equality across age cohorts, as were D_i_ and M_s_. Autoregressive pathway coefficients are listed in S1 Table. Asterisk indicates *P*<.01; double asterisks, *P*<.001; dagger, parameter constrained to 0; M_i_, mean intercept; D_i_, intercept variance (disturbance term); M_s_, mean slope; D_s_, slope variance.
(PDF)

S1 Table Autoregressive pathways from cohort sequential models. (PDF)

S1 Text Supporting information. (PDF)
