# Supplementary material for: The Avalanche Hypothesis and Compression of Morbidity: Testing Assumptions through Cohort-Sequential Analysis
Source: PLoS One. 2015 May 11;10(5):e0123910. doi: 10.1371/journal.pone.0123910 (PMC4427176; doi:10.1371/journal.pone.0123910)
Supplement: S1 Fig — Model fit was adequate (χ2(508) = 568.953, P = .03; χ2/df [NC] = 1.120; CFI = 1.000; RMSEA = .010). Note that a χ2/df ratio less than 2, a CFI greater than .95, and an RMSEA less than .06 all indicate that the model fits observed data adequately.1 Mi was constrained to equality across age cohorts, as were Di and Ms. Autoregressive pathway coefficients are listed in S1 Table. Asterisk indicates P<.01; double asterisks, P<.001; dagger, parameter constrained to 0; Mi, mean intercept; Di, intercept variance (disturbance term); Ms, mean slope; Ds, slope variance. (PDF) [file pone.0123910.s001.pdf]

**S1 Figure. Cohort-sequential model for annual healthcare costs.**

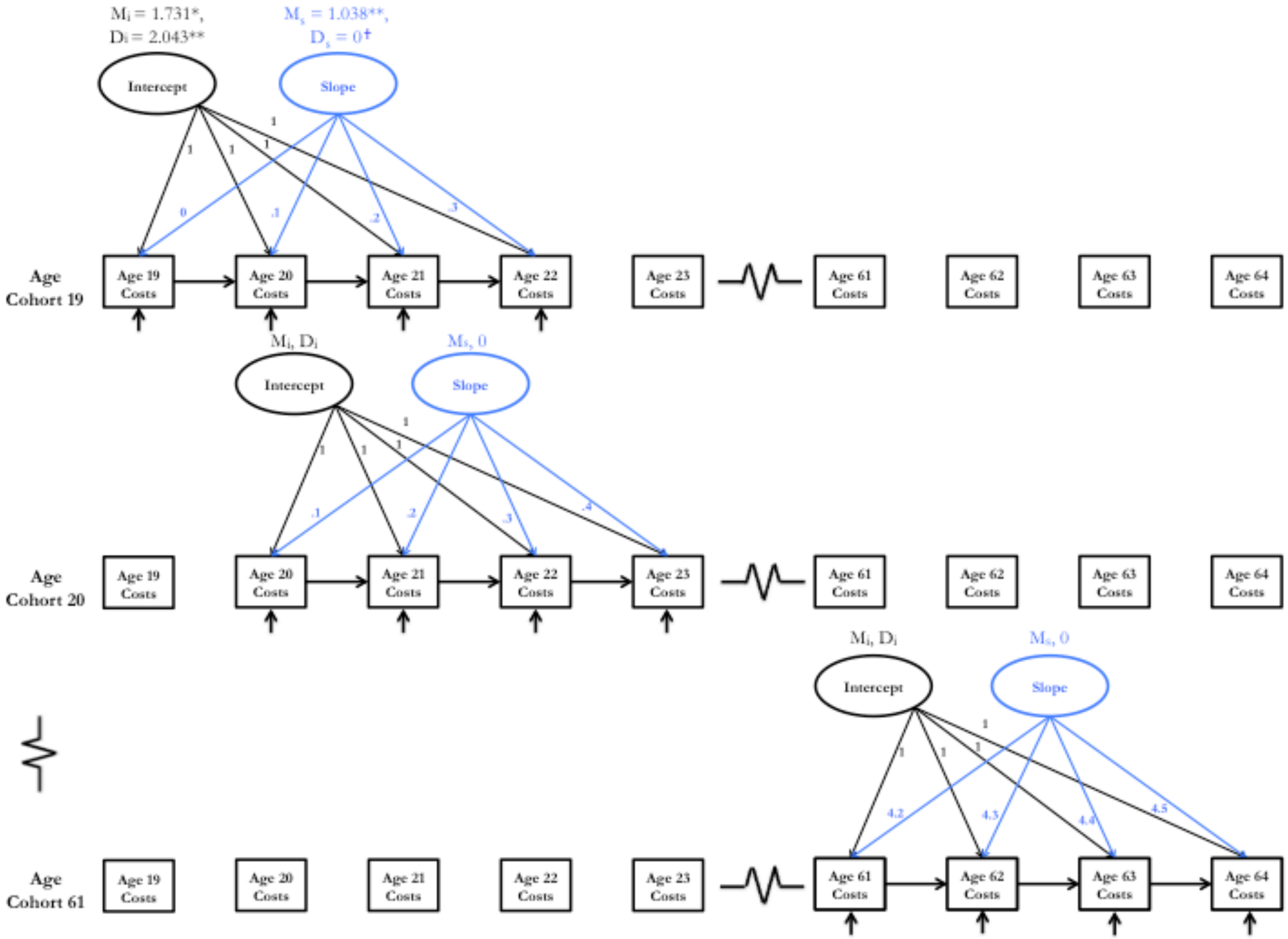


Model fit was adequate ($\chi^2(508) = 568.953$, $P$=.03; $\chi^2/df$ [NC] = 1.120; CFI = 1.000; RMSEA = .010). Note that a $\chi^2/df$ ratio less than 2, a CFI greater than .95, and an RMSEA less than .06 all indicate that the model fits observed data adequately.[1] $M_i$ was constrained to equality across age cohorts, as were $D_i$ and $M_s$. Autoregressive pathway coefficients are listed in S1 Table. Asterisk indicates $P$<.01; double asterisks, $P$<.001; dagger, parameter constrained to 0; $M_i$, mean intercept; $D_i$, intercept variance (disturbance term); $M_s$, mean slope; $D_s$, slope variance.

Reference

1. Tabachnick B, Fidell L. *Using Multivariate Statistics*. 6th ed. Upper Saddle River, NJ: Pearson; 2012: 74, 720-722.
