# Supplementary material for: The Avalanche Hypothesis and Compression of Morbidity: Testing Assumptions through Cohort-Sequential Analysis
Source: PLoS One. 2015 May 11;10(5):e0123910. doi: 10.1371/journal.pone.0123910 (PMC4427176; doi:10.1371/journal.pone.0123910)
Supplement: S1 Table — (PDF) [file pone.0123910.s002.pdf]

S1 Table. Autoregressive Pathways from Cohort Sequential Models

Autoregressive Pathway	Morbidity Score		Costs*	
	Autoreg. Coefficient (95% CI)	*P (two-tailed)*	*Autoreg. Coefficient (95% CI)*	*P (two-tailed)*
Age 19 → Age 20	-0.121 (-0.519 to 0.277)	.55	1.551 (-4.409 to 7.511)	.61
Age 20 → Age 21	0.179 (-0.080 to 0.438)	.18	1.039 (-0.178 to 2.256)	.09
Age 21 → Age 22	0.181 (0.004 to 0.357)	.05	0.873 (-2.213 to 3.958)	.58
Age 22 → Age 23	0.002 (-0.106 to 0.111)	.97	0.494 (-0.380 to 1.369)	.27
Age 23 → Age 24	0.087 (0.002 to 0.172)	.05	0.267 (0.000 to 0.534)	.05
Age 24 → Age 25	0.116 (0.046 to 0.186)	.001	0.369 (0.197 to 0.542)	<.001
Age 25 → Age 26	0.123 (0.061 to 0.185)	<.001	0.444 (0.297 to 0.590)	<.001
Age 26 → Age 27	0.089 (0.030 to 0.147)	.003	0.418 (0.287 to 0.549)	<.001
Age 27 → Age 28	0.161 (0.104 to 0.219)	<.001	0.414 (0.289 to 0.539)	<.001
Age 28 → Age 29	0.138 (0.082 to 0.194)	<.001	0.416 (0.303 to 0.529)	<.001
Age 29 → Age 30	0.154 (0.101 to 0.208)	<.001	0.439 (0.318 to 0.559)	<.001
Age 30 → Age 31	0.134 (0.082 to 0.186)	<.001	0.377 (0.279 to 0.476)	<.001
Age 31 → Age 32	0.192 (0.137 to 0.246)	<.001	0.485 (0.372 to 0.597)	<.001
Age 32 → Age 33	0.178 (0.129 to 0.227)	<.001	0.392 (0.296 to 0.489)	<.001
Age 33 → Age 34	0.120 (0.073 to 0.167)	<.001	0.351 (0.256 to 0.447)	<.001
Age 34 → Age 35	0.179 (0.134 to 0.224)	<.001	0.452 (0.356 to 0.548)	<.001
Age 35 → Age 36	0.164 (0.120 to 0.207)	<.001	0.372 (0.280 to 0.464)	<.001
Age 36 → Age 37	0.206 (0.164 to 0.248)	<.001	0.364 (0.275 to 0.454)	<.001
Age 37 → Age 38	0.179 (0.139 to 0.219)	<.001	0.369 (0.282 to 0.455)	<.001
Age 38 → Age 39	0.209 (0.166 to 0.251)	<.001	0.399 (0.304 to 0.494)	<.001
Age 39 → Age 40	0.202 (0.162 to 0.243)	<.001	0.391 (0.302 to 0.481)	<.001
Age 40 → Age 41	0.209 (0.169 to 0.249)	<.001	0.391 (0.298 to 0.484)	<.001
Age 41 → Age 42	0.200 (0.162 to 0.238)	<.001	0.427 (0.335 to 0.518)	<.001
Age 42 → Age 43	0.230 (0.191 to 0.268)	<.001	0.384 (0.283 to 0.485)	<.001
Age 43 → Age 44	0.211 (0.174 to 0.248)	<.001	0.327 (0.235 to 0.419)	<.001
Age 44 → Age 45	0.203 (0.167 to 0.239)	<.001	0.339 (0.244 to 0.434)	<.001
Age 45 → Age 46	0.196 (0.161 to 0.232)	<.001	0.349 (0.253 to 0.445)	<.001
Age 46 → Age 47	0.206 (0.171 to 0.241)	<.001	0.284 (0.185 to 0.383)	<.001
Age 47 → Age 48	0.205 (0.171 to 0.239)	<.001	0.314 (0.214 to 0.413)	<.001
Age 48 → Age 49	0.201 (0.168 to 0.234)	<.001	0.323 (0.228 to 0.418)	<.001
Age 49 → Age 50	0.200 (0.166 to 0.234)	<.001	0.285 (0.178 to 0.392)	<.001
Age 50 → Age 51	0.186 (0.153 to 0.219)	<.001	0.322 (0.220 to 0.425)	<.001
Age 51 → Age 52	0.229 (0.196 to 0.262)	<.001	0.336 (0.230 to 0.442)	<.001
Age 52 → Age 53	0.215 (0.181 to 0.248)	<.001	0.293 (0.178 to 0.408)	<.001
Age 53 → Age 54	0.232 (0.198 to 0.265)	<.001	0.295 (0.189 to 0.401)	<.001
Age 54 → Age 55	0.199 (0.166 to 0.232)	<.001	0.256 (0.145 to 0.368)	<.001
Age 55 → Age 56	0.215 (0.180 to 0.250)	<.001	0.235 (0.129 to 0.340)	<.001
Age 56 → Age 57	0.223 (0.187 to 0.259)	<.001	0.270 (0.168 to 0.372)	<.001
Age 57 → Age 58	0.246 (0.209 to 0.283)	<.001	0.270 (0.158 to 0.383)	<.001
Age 58 → Age 59	0.225 (0.188 to 0.262)	<.001	0.298 (0.178 to 0.419)	<.001
Age 59 → Age 60	0.176 (0.140 to 0.213)	<.001	0.174 (0.043 to 0.305)	.009
Age 60 → Age 61	0.169 (0.128 to 0.209)	<.001	0.176 (0.067 to 0.284)	.001
Age 61 → Age 62	0.179 (0.128 to 0.231)	<.001	0.170 (0.056 to 0.285)	.004
Age 62 → Age 63	0.227 (0.151 to 0.304)	<.001	0.180 (0.073 to 0.286)	.001
Age 63 → Age 64	0.063 (-0.014 to 0.141)	.11	0.062 (-0.049 to 0.174)	.27

*Costs were adjusted to 2009 values using the Consumer Price Indices from the U.S. Bureau of Labor Statistics.[1]

Reference

1. U.S. Department of Labor, Bureau of Labor Statistics. All urban consumer not seasonally adjusted: US city average. 2009. Available at: http://data.bls.gov/PDQ/outside.jsp?survey=cu. Accessed August 22, 2012.
