# Supplementary material for: The Avalanche Hypothesis and Compression of Morbidity: Testing Assumptions through Cohort-Sequential Analysis
Source: PLoS One. 2015 May 11;10(5):e0123910. doi: 10.1371/journal.pone.0123910 (PMC4427176; doi:10.1371/journal.pone.0123910)
Supplement: S1 Text — (PDF) [file pone.0123910.s003.pdf]

Supporting Information for:

The Avalanche Hypothesis and Compression of Morbidity: Testing Assumptions through Cohort-Sequential Analysis

Jordan Silberman, PhD; Chun Wang, MS; Shawn Mason, PhD; Steven M. Schwartz, PhD; Matt Hall, BS; Jason L. Morrissette, MA; Xin M. Tu, PhD; and Janet Greenhut, MD, MPH

Supplementary Methods

Was the Counterintuitive Order of Tipping Points due to Costs of Preventive Health Services?

To potentially rule out preventive health services as a driver of the costs tipping point, we used a multiple-step procedure similar to the one used for estimation of the hockey stick regression models described in the main text. First, we regressed total costs on age and outpatient costs, separately for each subject. Next, we estimated a piecewise regression model in which subject-specific unstandardized age coefficients from the first step were specified as the dependent variable, and subjects' mean ages during the 4-year study period were specified as the independent variable. A knot was fixed at 39.5 years of age. FGNLS estimation was used for reasons described in the main text. We then estimated a second piecewise regression that was identical to the first, except that the coefficients specified as the dependent variable were those computed without controlling for outpatient costs.

Finally, we tested the hypothesis that the costs tipping point would be attenuated after controlling for outpatient costs. Stata's **nlsur** and **lincom** commands were used to compare the difference between pre- and post-tipping point slopes observed in the uncontrolled piecewise regression to that of the controlled piecewise regression. The method used to test the significance of this difference between differences is described elsewhere [1], and Stata syntax is available upon request from the first author.

Supplementary Results

The difference between pre- and post-tipping point slopes observed in the uncontrolled model did not differ from that of the controlled model ("$\Delta\Delta b$"=0.385; 95% CI, -14.314-15.084; P=.96). This suggests that controlling for outpatient costs did not attenuate the costs tipping point. As explained above, if preventive services at least partially accounted for the observed costs tipping point, then controlling for outpatient costs should have attenuated the costs tipping point. Thus, results suggest that the costs tipping point observed at 39.5 years of age cannot be attributed to changes in preventive health services.

Reference

1. StataCorp, LP. Stata 12 Base Reference Manual. College Station, TX: Stata Press; 2011: 916-917, 2179.
